# Flow experience in foreign language writing: Its effect on students’ writing process and writing performance

**DOI:** 10.3389/fpsyg.2022.952044

**Published:** 2022-08-04

**Authors:** Ping Liu, Yao Zhang, Dilin Liu

**Affiliations:** ^1^School of Foreign Languages, Southeast University, Nanjing, China; ^2^Fuzhou Pingdong Secondary School, Fuzhou, China; ^3^Department of English, University of Alabama, Tuscaloosa, AL, United States; ^4^School of English Studies, Dalian University of Foreign Language, Dalian, China

**Keywords:** flow, second language writing, positive psychology, motivation, attention

## Abstract

This research investigates Chinese EFL students’ flow experience in academic writing and its effect on students’ writing performance. The research consists of two studies: (1) a preliminary study involving a survey of 162 college students immediately after their completion of a short English essay to examine whether and how intensely they experienced flow during their writing and whether their perceived levels of challenge of the writing task and their writing skills affected their flow experience, and (2) a main study including a survey of 216 different students at the end of a semester-long writing course to ascertain how frequently these students experienced flow during the course, whether their intrinsic writing motivation and attention control ability were significantly correlated with their flow experience, and whether their flow frequency had an effect on their performance in the writing course. Results of statistical tests (including Class Factor Analyses and regression analyses) of the data in the preliminary study revealed that a large majority (76%) of the students experienced a certain level of flow in their writing and their perceived levels of writing skills had a significant influence on their flow experience. The statistical test results of the main study indicated that (1) 66.4% of the students experienced flow with various frequency levels, while 33.6% of the students rarely or never experienced flow, (2) students’ level of motivation and attention control were significantly correlated with their level of flow frequency, and (3) students’ flow frequency had a significant influence on their writing course scores. Research and pedagogical implications of the study are also discussed.

## Introduction

Two recent systematic reviews (Pelaez-Morales, 2017; Riazi et al., 2018) of the research articles published in *Journal of Second Language Writing* (*JSLW*) since the journals’ inception in 1992 indicate that research on second language (L2) writing has grown significantly both in amount and scope, especially in the past decade. Pelaez-Morales (2017, p. 18) uncovered a total of 26 main research topics, including “feedback, genre, assessment, lexis, syntax, and voice,” and “sociopolitical/cultural issues” while Riazi et al. (2018, p. 47) identified 10 research foci (with quite a few of them overlapping with Pelaez-Morales’ uncovered topics), including “feedback, instruction, language and literacy development,” and “assessment.” Riazi et al. (2018, p. 41) also uncovered a range of “main theoretical orientations” in L2 writing research: “cognitive, social, socio-cognitive, genre, contrastive rhetoric, and critical theories.” However, surprisingly, psychology or psychology-related issues failed to make these lists of main research topics/foci in L2 writing, which seems to suggest that there is a lack of enough psychology-oriented research on L2 writing. Of course, the fact that psychology-related topics did not make these lists of research foci in L2 writing does not mean that there has not been research on these issues. In fact, there have been quite a few L2-writing studies dealing with psychological/sociopsychological issues, such as anxiety, attitude, motivation, self-efficacy, and self-regulation (Hashemian and Heidari, 2013; Ringel, 2014; Waller and Papi, 2017; Rahimi and Zhang, 2018; Payant and Zuniga, 2022). These studies have produced important findings about how positive psychological factors (such as positive attitude and strong motivation) may enhance L2 writing performance and development and how negative psychological factors (such as high anxiety and low or lack of motivation) may hinder L2 writing performance and development.

Yet, it is interesting to note that only two of these psychology-related studies on L2 writing (Ringel, 2014; Payant and Zuniga, 2022) seemed to have been carried out explicitly in the theoretical framework of positive psychology, a fact that is quite puzzling considering that the past 2 decades have witnessed a significant increase in the number of positive psychology-guided studies in the field of foreign/second language learning/acquisition or SLA, to use a more generic acronym for simplicity purposes, (e.g., Egbert, 2003; Kirchhoff, 2013; Gabryś-Barker and Gałajda, 2016; MacIntyre, 2016; Aubrey, 2017a,b; Piniel and Albert, 2017; Dewaele et al., 2019). These studies in SLA have covered various aspects of positive psychology, including emotion (e.g., Dewaele, 2005, 2015; Saito et al., 2018), flow experience (Egbert, 2003; Czimmermann and Piniel, 2016; Aubrey, 2017a,b), and motivation (Piniel and Albert, 2017; Saito et al., 2018). They have also produced results that enhanced our understanding of the role of positive psychology in SLA, signaling the potential and need for more such research in SLA (Dewaele et al., 2019; MacIntyre et al., 2019), especially in L2 writing where, as noted earlier, it is lacking.

Ringel (2014) and Payant and Zuniga (2022), the only two aforementioned positive-psychology-guided studies on L2 writing, examined L2 learners’ flow experience in writing classes. Flow experience, a construct or theory in positive psychology, refers to an optimal state or experience of being completely engaged in an enjoyable and interesting activity (Bempechat and Shernoff, 2012). It has been found to yield an important positive effect on SLA in various learning activities and in learning some specific languages skills, such as in classroom interactive activities (Egbert, 2003; Aubrey, 2017a,b; Payant and Zuniga, 2022) and in extensive reading (Kirchhoff, 2013; Zare-ee, 2013). Given the importance of flow experience in SLA and a lack of enough research of it in L2 writing, the present research aims to examine flow experience in Chinese college EFL writers as well as factors affecting their flow experience and the effect of flow experience on their writing performance. The rest of the article is organized as follows: a brief review of relevant research on positive psychology and flow experience in SLA and L2 writing; a description of the research including its overall design and the two survey studies conducted, covering research questions, methodologies, and data analyses as well as the reporting and discussion of the results; and a conclusion.

## Literature review

### Positive psychology and its role in SLA

The modern concept of positive psychology was developed by M. E. P. Seligman and M. Csikszentmihalyi in the late 1990s and the early 2000 to help investigate how average humans thrive and flourish (Seligman and Csikszentmihalyi, 2000). This theory focuses on three key aspects: “positive subjective experience, positive individual traits, and positive institutions (Seligman and Csikszentmihalyi, 2000, p. 5). Positive psychology distinguishes itself from general psychology by concentrating on the positive sides of human life instead of the negative facets of life and the belief that people can embrace happiness by promoting human advantages and strengths (Seligman and Csikszentmihalyi, 2000, 2014; Mercer and MacIntyre, 2014). Besides, positive psychology has been found to promote instantaneous thinking and cultivate creative thinking and action (Gregersen, 2016).

In the field of SLA, research has explored and shown specifically how positive psychology can help improve individual learners’ emotional experience and enhance their happiness in foreign language learning, which would in turn help build an encouraging foreign language classroom environment (Dewaele, 2005, 2015; MacIntyre and Gregersen, 2012; MacIntyre, 2016; Dewaele et al., 2019; MacIntyre et al., 2019). Research on positive psychology interventions in L2 learning has also been carried out to help students and teachers develop and strengthen their courage, creativity, happiness, optimism, and overall wellbeing for more effective language learning and such research has flowered since 2016 (Dewaele et al., 2019). It has been found that positive psychology can help make L2 learners move “from negative to positive emotion,” “from deficiencies to strengths,” and “from Positive Emotion, Engagement, Relationships, Meaning, and Achievement (PERMA) to Emotion/Empathy, Meaning/Motivation, Perseverance including resilience, Agency/Autonomy, Time, Habits of mind, Intelligences, Character strengths, and Self-factors, especially self-efficacy (EMPATHICS),” hence culminating in “moving into flow” (MacIntyre, 2016, p. 6–9).

### Flow experience and its role in SLA

Flow, an important theory in positive psychology developed by Csikszentmihalyi (1975), refers to an optimal state or experience of being completely engaged in an enjoyable and interesting activity, in which, “people are so involved in an activity that nothing else seems to matter; the experience itself is so enjoyable that people will do it even at great cost, for the sheer sake of doing it” (Csikszentmihalyi, 2008, p. 4). In this psychological state, individuals may hardly notice the lapse of time, their self-consciousness may fade away, and their competence can maximize (Seligman, 2012; Nakamura and Csikszentmihalyi, 2014). Research has found that individuals who reportedly have a higher level of flow experience in a given activity tend to practice it more frequently, spend more time doing it, and perform it better (O’Neill, 1999). Besides, “experiencing frequent flow states within a specific activity leads to a desire to perform that activity for its own sake,” which means the activity becomes autotelic (Jackson et al., 1998, p. 359). Furthermore, a continuous flow experience is thought to not only increase individuals’ intrinsic interest in activities, but also show an increasing effect on positive emotions (Li et al., 2019; Özhan and Kocadere, 2020).

As expounded by Csikszentmihalyi (1990, p. 49-67) and summarized by Jackson (1996, p. 77), the characteristics of flow fall into nine main dimensions (listed in Table 1). Based on the information in the table, a person who experiences flow in an activity typically displays a strong concentration on the task at hand; enjoys an altered sense of time, a loss of consciousness, and a sense of control with no worry of failure; demonstrates skills that match the challenges of the task; possesses clear goals; and knows how well the activity is going. All of this helps make the activity being performed totally autotelic, giving the person “an intrinsically rewarding experience involving a sense of deep enjoyment” (Jackson, 1996, p. 77). Finally, it is necessary to explain that the “unambiguous and immediate feedback” dimension does not refer to feedback from one’s instructor or peers. Rather it refers to what the person feels how the activity is going based on what he gathers from the ongoing activity itself and any other useful contextual information.

**Table 1:** Dimensions of flow experience.

Concentration on the task at hand
Transformation of time (i.e., altered sense of time)
Matched skills and challenges (i.e., one’s skills match the challenges of the task)
Clear goals
Merging of action and awareness
Loss of self-consciousness
Sense of control
Unambiguous and immediate feedback (i.e., knowing how well the activity is going)
Autotelic experience (i.e., the activity/task becomes autotelic)

Many studies have confirmed the effectiveness of using these nine dimensions to help identify and measure flow experience and have included them in the development of instruments for measuring flow experience in sports activities (e. g., Jackson and Eklund, 2002; Jackson et al., 2008). Some of the dimensions have also been used in the flow measurements in L2 learning research (Egbert, 2003; Aubrey, 2017a,b).

Regarding research on flow experience in SLA, existing studies have confirmed the existence of such experience among L2 learners during various classroom activities (Egbert, 2003; Kirchhoff, 2013; Zare-ee, 2013; Czimmermann and Piniel, 2016; Aubrey, 2017a,b). Egbert (2003, p. 499) studied the experience of American college students in a Spanish class doing specially designed online chatting/email exchange tasks and the results showed that these learning tasks engendered flow experience. Egbert (2003) believes her findings suggest that “Flow Theory offers an interesting and useful framework for conceptualizing and evaluating language learning activities” (Egbert, 2003, p. 499).

The case study of Kirchhoff (2013) on flow experience in EFL extensive reading reports that students did experience flow while reading. Similarly, research of Zare-ee (2013) on flow experience and EFL reading shows that flow experience can increase students’ interest in reading and help them better understand the texts when the reading tasks are appropriate (Zare-ee, 2013). Furthermore, study of Mcquillan and Conde’s (1996) on both L1 and bilingual speakers’ reading experience also finds that flow occurs for both L1 and L2 reading when participants read for pleasure and texts are attractive and intrinsically beneficial.

Flow experience has also been found to be positively related to L2 learning attitude and motivation. For example, Piniel and Albert (2017, p. 100) report that “[i]nducing flow experiences can lead to a higher level of language learning motivation.” Studies (Shernoff et al., 2003; Liu and Song, 2021) have also revealed that students’ attitude toward language learning may change through facilitating flow experience in the classroom.

However, even though flow experience has drawn attention from researchers and has been promoted for helping improve positive experience in various L2 learning contexts and in learning various L2 skills, only two studies (Ringel, 2014; Payant and Zuniga, 2022), as noted above, have investigated flow experience in L2 writing. Payant and Zuniga (2022) investigated the flow experience of L2 learners (“additional language learners” in their term) during peer revisions (two rounds) in an online writing course. The results of the students’ response in the perceived flow experience survey (given after each peer revision task on the same writing assignment) indicate that “engaging learners in the same tasks on two occasions appeared to be flow-inducing,” leading to “an increased perception of focus and SCB (Payant and Zuniga, 2022, p. 8). This is because doing the same task the second time enabled learners to gain “familiarity of task expectations and technologies” and appreciate “opportunities to exchange and collaborate” (Payant and Zuniga, 2022, p. 8). Ringel (2014, p. viii) used semi-structured interviews, flow experience surveys, and students’ journals to examine whether and how the use of one’s L2 might affect ESL writers’ flow experience and to explore “how multimodal and multiliteracy assignments may allow greater possibilities for flow” in an ESL composition class of 25 students. The results of the data analysis indicated that “flow states were very difficult [for L2 writers] to achieve while working on traditional writing assignments” but were present when they were “conducting research in their area of interest,” pointing to L2 writers’ interest as a key (Ringel, 2014, p. 59–60). The results of the study also show multimodal writing tasks (i.e., those that involve not only the use of words but also visuals) that engage multi-literacies (e.g., computer literacy and students’ L1 literacies) were more likely to engender flow experience than conventional mono-modal and mono-literacy writing tasks.

While these two studies have produced some interesting findings about L2 learners’ flow experience in writing, they were limited in several aspects. First, neither study investigated the effect of flow experience on L2 learners’ writing performance. Second, the sample size in both studies was small (18 and 25 students, respectively). Third, the survey instruments used in both studies covered only a few (four or five) of the nine dimensions of flow mentioned by Csikszentmihalyi (1990) and Nakamura and Csikszentmihalyi (2014) and included in the frequently used flow measurement scales, such as those developed by Jackson and Eklund (2002) and Jackson et al. (2008). Fourth, the existing studies did not specifically examine how flow experience is related to l2 writers’ psychological states during the writing process and how their perceived level of difficulty and their perceived writing skills affect their flow in L2 writing. Overall, while understanding flow in L2 writing is extremely important for L2 teachers in their effort to enhance L2 learners’ wiring experience and performance, so far, there have been only two studies that directly examined this topic. Furthermore, these two existing studies, as just mentioned, are limited in several important aspects, i.e., they have left several gaps in the existing research on flow in L2 writing. It seems pivotal for research on flow in L2 writing to examine the relationship between L2 writers flow experience and their psychological states during writing and to investigate the effect of flow on L2 learners’ writing performance, especially by using a large sample size and a more complete flow measurement instrument. Against this backdrop, this research aims to use large samples to investigate flow experience in college EFL writing, its relationship with EFL students’ other psychological states concerning writing, and its effect on these students’ writing performance.

## Overall research design and research questions

This research consisted of two studies, a preliminary study and a main study, with each focusing on different issues related to flow experience in L2 writing. The preliminary study was so called because it was conducted to simply determine or identify whether EFL learners would experience flow in the writing of an essay and, if identified, how intense this experience would be. Positive findings from this preliminary study would justify and lead to our main study, which was intended to examine three issues related to flow in EFL writing: (1) the frequency with which EFL learners would experience flow in writing during a semester-long writing course, (2) the relationship between their flow frequency and their other psychological states, including perceived intrinsic motivation and attentional control, and (3) the effects of flow frequency on students’ writing performance. Both studies were conducted in the same semester (with the preliminary study at the middle and the main study at the end of the semester) and the participants of both studies were non-English major students enrolled in English writing classes at a large university in China. To avoid any undue influence from the preliminary study on the results of the main study, a different group of students were recruited as participants for the main study. This research aimed to answer the following research questions, with Questions 1 and 2 for the preliminary study and Questions 3, 4, and 5 for Study 2:

Q1: Whether and how intensely do EFL students experience flow in writing?
Q2: What is the relationship between EFL students’ perceived level of challenges of the writing task and their own perceived writing skills on the one hand and their flow experience on the other?
Q3: Whether and how frequently do EFL students experience flow in writing across a semester-long writing course?
Q4: What is the relationship between EFL students’ flow experience and their perceived level of intrinsic motivation and attention control?
Q5. What is the effect of students’ flow frequency on their writing performance?

It is crucial to note that research questions 2–5 specifically address the gaps in the existing research discussed above. Below we first describe the methodology and report the results of the preliminary study and then those of the main study.

## Preliminary study

### Methodology

#### Participants

About 162 non-English major sophomores (109 females and 53 males) enrolled in three sections of the writing course titled “IELTS [a British English proficiency test] Writing” at the aforementioned Chinese university voluntarily participated in this study after being informed of the study and filling out a consent form. Specifically, one of the researchers went to the three classes, explained to the students what the survey study was about and what participants would need to do as well as the fact that participation was voluntary, and participants were able to withdraw from the study anytime. The participants were all native speakers of Chinese and had studied English for at least 9 years by the time of the study. These students were taking the IELTS writing class not only because it was one of the courses that they could take to meet their writing course requirement for graduation but also because they needed to enhance their writing skills to pass the College English Test 4 (CET-4), a national English proficiency test with a writing component that all college students in China must take and pass to graduate. In short, the reasons these students were recruited for this study were twofold. First, they were enrolled in an academic writing course and hence were actively engaged in learning academic writing, a *de facto* requirement for participation in this survey study. Second, as just explained, these students appeared to have both the motivation and need to learn L2 writing, a fact that made them good candidates for participating in the study.

#### Task and instrument used

##### Writing task

This task asked the participants to compose an essay with a minimum of 200 words based on the prompt: “If you are given a million dollars to spend anything want, explain how you will spend the money and why.” This essay simulated the writing task in the Chinese CET-4, a national English competence test that Chinese university students were required to take and pass.

##### Questionnaire

This was a two-part questionnaire (see Appendix 1). Part 1 consisted of two 10-point Likert scale questions (with 1 being “Extremely low” and 10 being “Extremely high”) with Question 1 asking students to rate the difficulty level of the writing task they just completed, i.e., the aforementioned writing task, and Question 2 requesting them to rate their own writing skills displayed in performing the writing task. The two questions were adapted from two similar questions in the instrument that Jackson et al. (1998) used in a study on psychological correlates with flow experience in sports for accessing participants’ perceived challenges of the sports activity performed and their skills in performing the activity. For easier reference, we label this part of the questions as Perceived Writing Challenge and Skill Scale (*PWCSS*). The results of this scale are intended to be used to help determine how the students’ perceived challenge level of the writing task and their own perceived level of writing skills in the performance of the task might influence or predict their flow experience in the writing task.

Part 2 included nine five-point Likert scale questions (with 1 being “Strongly disagree” and 5 being “Strongly agree”) designed for ascertaining whether and how intensely the students experienced flow when doing the writing task. Each of the nine questions dealt with one of the nine established dimensions of flow (see Table 1 and Appendix 1 concerning the nine dimensions). For example, Question 1 in this part asked the students to rate the statement “While writing the essay, I was totally concentrated on the writing task, not at all distracted,” a question covering the “concentration on the task at hand” dimension of flow. The nine flow-related questions were adapted from the nine questions in short form of the *Flow State Scale-2* (*FSS-2*) of Jackson et al. (2008). The long form of the *FSS-2* had 36 questions and it was enhanced version of the original *FSS* of Jackson and Eklund (2002) develop by Jackson and Marsh (1996) for assessing the intensity of participants’ flow experience in sports activities, with four question items for measuring each of the nine flow dimensions (4 questions in each dimension × 9 dimensions = 36 total items). The nine-item short form of *FSS-2* included one representative question from each of the nine dimensions in the long form. Jackson et al. (2008) compared the short form with the long form in terms of effectiveness, validity, and reliability. Their results show that the short form “may provide an opportunity to assess the experience without imposing too many restrictions on measurement of more central constructs” and appears as reliable and valid as the long form (Jackson et al., 2008, p. 563). In adapting the nine questions from Jackson et al.’s (2008) short form of *FSS-2*, we carefully changed the wording and context from sports to English writing. For example, our Question 1 “While writing the essay, I was entirely focused on the writing task without feeling any distraction” was adapted from Jackson et al.’s (2008, p. 567) “I was completely focused on the task at hand.” To differentiate our flow scale from Jackson et al.’s (2008) *FFS* for sports, we label it Writing Flow State Scale (*WFSS*).

### Data collection procedure and data analysis

The 162 students were first asked to write an essay with at least 200 words within 45 min on the topic mentioned in the Writing Task section above at the middle the fall semester. Immediately after finishing the essay, the students were requested to complete the aforementioned *WCSSS* and *WFSS* questionnaire online in 15 min, using mobile devices. It is important to note that although 162 students participated in this study, 27 students’ data were excluded for analysis because these students either chose the same answer for all the questions in the questionnaire or employed the same answer pattern, e.g., a–d; a–d.

All the data collected in this research were analyzed by using SPSS 24.0 (Statistical Package for the Social Science 24.0) and LatentGold v.5.0.0, a statistical program for doing Latent Class Factor Analysis (LCFA) to be explained below. Given there were nine questions in the *WFSS*, we first ran a Cronbach’s Alpha test of this scale to determine its internal consistency reliability. The test result (Alpha value being 0.809) indicates that the *WFSS* had acceptable reliability.

To answer research question 1, we performed an LCFA, which is a statistical procedure developed by Kawabata and associates (Kawabata and Mallett, 2012; Kawabata and Evans, 2016) for the purpose of identifying and classifying different levels of flow intensity and frequency. According Kawabata and associates, a score above three (out of the five total) for each of the nine dimensions of flow in the *FSS* indicates that flow experience occurred (Kawabata and Mallett, 2012). LCFA has been found to be able to successfully differentiate flow experiencers from non-flow experiencers (Kawabata and Evans, 2016) and identify those who experienced flow state more frequently from those who did less (Kawabata and Mallett, 2012). Therefore, we decided to adopt the LCFA procedure in measuring flow state intensity in L2 writing in this preliminary study and flow frequency in the main study. As noted above, our LCFAs of the questionnaire data were conducted by using the LatentGold v.5.0.0 program (Vermunt and Magidson, 2013). To answer Research Question 2, we ran a regression test to determine whether and how the students’ perceived challenge level of the writing task and their own perceived level of writing skills in the performance of the task might predict their perceived flow experience (intensity) during the writing task.

### Results and discussion

The LCFA we performed first produced goodness-of-fit statistics showing various possible groupings (models) of the students based on their response scores on the *WFSS*. The model statistical results (reported in Table 2) include seven models for grouping the participating students (i.e., the students grouped in seven different models, ranging from a one-group model to a seven-group model). The reason we included seven models was that previous research included six models (Kawabata and Mallett, 2012) so we added one more model to help ensure that the best model could be identified. To determine which grouping model was the best fit, we need to look at the Bayesian Information Criterion (BIC) values (Kawabata and Mallett, 2012). Research has shown that the model with the lowest BIC value is considered the best fitting model (Kawabata and Mallett, 2012). As shown in Table 2, the lowest value of BIC appeared in model 3. Therefore, model 3 (i.e., the three-group model) was the best fit. In other words, the three-group model most accurately represented the distributions of the subjects based on their *WFSS* response patterns, i.e., their scores in the nine flow dimensions to be specific.

**Table 2:** Goodness-of-Fit statistics for Latent Class Factor Analysis (LCFA) Models on Writing Flow State Scale (*WFSS*; *n* = 135).

Model	LL	BIC(LL)	Npar	df	*p* value
1 Group	−1463.005	3078.073	31	104	1.40e−274
2 Groups	−1367.165	2935.445	41	94	1.40e−241
3 Groups	−1326.944	2904.056	51	84	3.30e−231
4 Groups	−1302.925	2905.072	61	74	1.50e−227
5 Groups	−1285.379	2919.032	71	64	9.60e−227
6 Groups	−1268.575	2934.476	81	54	1.80e−226
7 Groups	−1246.67	2939.721	91	44	2.20e−224

After it was determined that the three-group model was the best fit, we were then able to find out the three groups’ response pattern profiles. The LCFA yielded the following types of information regarding the groups’ profiles: (1) the percentage of each of the three groups in the total number of participants, (2) each group’s mean scores for the perceived challenge level of the writing task and perceived level of writing skills displayed in the writing task, and (3) each group’s mean score in each of the nine dimensions of flow experienced measured by the *WFSS.* All three types of information are reported in Table 3. It is important to note that the groups are numbered in order of their percentage sizes with Group 1 boasting the largest percentage.

**Table 3:** Profile of three-group LCFA Model of *WFSS.*

Category	Subcategory/dimensions	Mean score		
		Group 1	Group 2	Group 3
Percentage		0.5890	0.2396	0.1714
*PWCSS*	Writing challenge level	5.902	6.806	5.909
	Writing skill level	5.427	4.452	5.591
*WFSS*	Concentration	3.804	3.092	4.345
	Transformation of time	3.897	3.785	4.870
	Challenge-skill balance	3.433	2.996	4.372
	Clear goals	3.597	2.935	4.367
	Action awareness merging	3.127	2.254	3.735
	Loss of self-consciousness	3.576	2.782	4.180
	Sense of control	3.388	2.344	3.966
	Unambiguous feedback	3.806	3.152	4.467
	Autotelic experience	3.752	3.200	4.674
	**Overall mean**	**3.598**	**2.949**	**4.331**

The bold figures are the total numbers.

**Figure 1:**
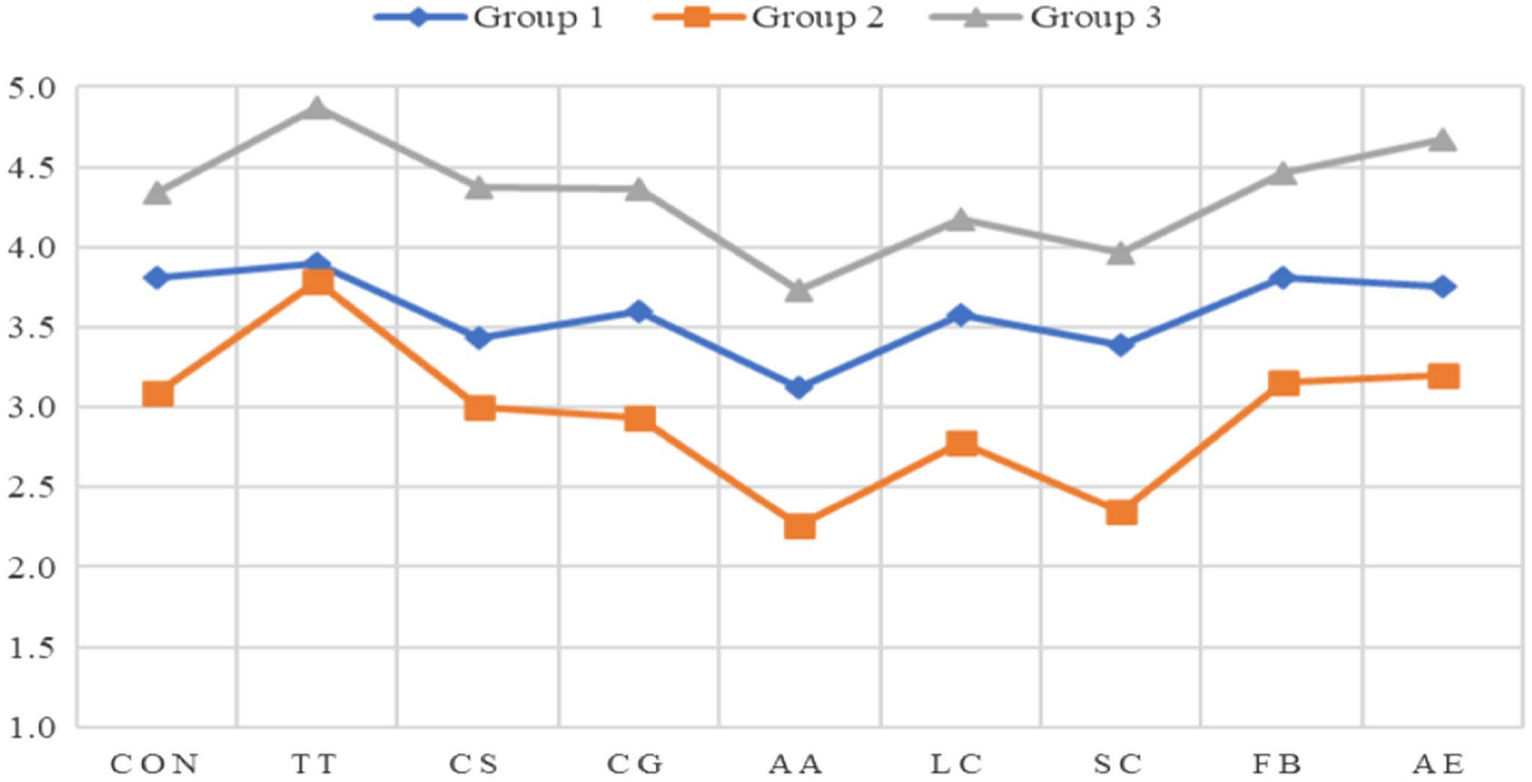
Profile plot of the Three-Group LCFA Model of *WFSS*. CON, concentration on the task at hand; TT, transformation of time; CS, challenge-skill balance; CG, clear goals; AA, action-awareness merging; LC, loss of self-consciousness; SC, sense of control; FB, unambiguous feedback; and AE, autotelic experience. The *y*-axis listed item-average scores and the *x*-axis presented flow dimensions.

To help us visualize and better understand the flow state profiles of the three groups, a profile plot is presented in Figure 1 based on the three groups’ mean flow state scores in the nine dimensions reported in Table 3. The *x*-axis represents the nine flow dimensions, and the *y*-axis lists the groups’ mean scores of those dimensions. As can be seen in the plot in Figure 1, the three groups were separated from each other without any entanglement. For Group 1 and Group 3, the mean scores of all the flow dimensions and their overall mean were all above 3.0, the point of the *WFSS* that, as mentioned earlier, indicates that flow experience occurred (Kawabata and Mallett, 2012). Also, according to Kawabata and Evans (2016), the mean scores in three of the dimensions, including the balance between challenges and skills, clear goals, and unambiguous feedback, should adopt a more stringent or higher point of 3.4 instead of 3.0 to indicate the occurrence of flow. The mean scores of both Groups 1 and 3 in these three dimensions were all above 3.4. In short, based on all the established criteria established in previous research (Kawabata and Mallett, 2012), both Groups 1 and 3 clearly experienced flow with Group 3 showing a much higher intensity of flow than Group 1 as evidenced by its higher mean scores. In contrast, Group 2 did not experience flow as evidenced by the fact that its overall mean score was under 3 and its mean scores were under three in five (a majority) of the nine dimensions (challenge-skill balance, clear goals, action-awareness merging, loss of self-consciousness, and sense of control). Even in the four dimensions where its scores were above 3, the scores were substantially lower than those of Group 1 and Group 3, indicating a much lower flow intensity.

Another crucial point to note is that Group 1 and Group 3, who experienced flow, accounted for a combined 76% (59 + 17%) of the total number of the participating students while Group 2 accounted for only 24%. In other words, an overwhelming majority of the students experienced flow. Yet it is also simultaneously important to point out that of the 76% who experienced flow, 59% (an overwhelming majority) showed relatively low flow intensity (with their mean score being 3.598). Only 17% boasted high flow intensity (with their mean score being 4.331).

Furthermore, as noted above, Table 3 also includes the mean scores of each group’s perceived challenge level of the writing task and perceived level of writing skills in the performance of the task Clearly, Group 1’s and Group 3’s perceived challenge levels (5.902 and 5.909, respectively) were much lower than Group 2’s (6.806) while the former two’s perceived levels of writing skills (5.427 and 5.591) were much higher than Group 2’s (4.452). These results seem to suggest that Group 1 and Group 2’s lower perceived challenge levels but higher perceived writing skill levels might have contributed to their higher flow intensity levels while Group 2’s higher perceived challenge level and lower perceived writing skill level might have led to their lack of flow experience in a majority of the nine dimensions and a lower flow intensity in the four dimensions they showed flow experience. To help ascertain whether this was indeed the case, i.e., to determine whether students’ perceived writing challenge levels and writing skill levels would influence or predict their flow experience, we ran a multiple regression test.

The results of the multiple regression test show the two predictors (students’ perceived level of challenges of the writing task and their perceived level of writing skills displaced during writing) explained 19.1% of the variance in their flow experience [*R^2^* = 0.191, *F* (2, 132) = 15.563, *p* < 0.000]. It was found that the students’ perceived level of writing skills significantly predicted their flow experience (*β* = 0.151, *p* < 0.000, see Table 4 for more detail) while their perceived level of challenges of the writing task did not significantly (although almost significantly) predicted their flow experience as well (*β* = −0.043, *p* < 0.085). In other words, generally, when students’ perceived level of skills in writing increased and (to a lesser degree, their perceived level of the challenges of the writing task decreased), their flow experience increased.

**Table 4:** Estimates of the multiple regression test.

Source	*B*	*SE*	*β*	*t*	*p*
Constant (Intercept)	3.042	0.246		12.372	0.000
Perceived challenges	−0.043	0.025	−0.138	−1.733	0.085
Perceived skills	0.151	0.031	0.388	4.853	0.000

In summary, the results of the preliminary study show that flow experience did occur for most of the EFL students during the writing task and students’ perceived level of writing skills significantly affected their flow experience and their perceived level of challenges of the writing task almost had a significant effect on their flow experience.

## Main study

### Methodology

#### Participants

About 216 non-English major sophomores (148 females and 68 males) from the same university mentioned above participated in this study after being informed of the study and filling out a consent form. The demographics of these 216 students were identical to those of the participants in the preliminary study except that these students were all enrolled in four sections of a different writing course titled English Academic Writing. The reasons that these 216 students were recruited for this main study were the same as those for recruiting the 162 participating in the preliminary study.

#### Task and instrument used

While no specifically assigned writing task was involved for the sake of this study, the participants were asked to respond to the questions in the questionnaire based on their writing experience throughout the writing course they had just completed. Furthermore, the students’ final grades or scores in the writing course were used as the measurement of their writing performance in the course. The full course score was 100, 75% of which was the score of the course research paper and 25% was the score of an essay critiquing and evaluating the research paper.

#### Questionnaire

This was a three-part questionnaire (see Appendix 2). Part 1 was almost identical to Part 2 (the *WFSS*) used in the preliminary study except for the following two differences. First, the participants were asked to answer the questions based on their writing experience in the writing course throughout the semester they had just completed, rather than based on a writing task completed immediately before answering the questionnaire as was the case in the preliminary study. Second, instead of answering whether and how intensely they experienced flow, the participants were asked to indicate how frequently they experienced each of the nine dimensions of flow, with 1 meaning Never and 5 indicating Always. This part of the questionnaire is labeled Writing Flow Frequency Scale (*WFFS*).

Questions in Parts 2 and 3 were identical in format as those in Part 2: all five-point Likert scale questions. Part 2 included 11 questions designed to measure the students’ perceived intrinsic motivation/amotivation for writing. These questions were adapted from three different sources: questionnaire of Noels et al. (2001) on L2 intrinsic motivation and Scale of L2 Intrinsic Motivation of Wu (2003). The 11 questions covered three aspects of intrinsic motivation: amotivation, intrinsic motivation to gain knowledge and stimulation (knowledge-stimulation), and intrinsic motivation to feel accomplished (accomplishment), with Questions 3, 7, 10, and 11 on amotivation, Questions 1, 4, and 8 on knowledge-stimulation, and Questions 2, 5, 6, and 9 on accomplishment. This part of the questions was labeled Intrinsic Motivation Scale (IMS). It is necessary to note that some of the IMS questions (Questions 4–7) were negatively states, e.g., Part 3 of the questionnaire consisted of nine questions developed to measure the students’ perceived attentional control in writing. These questions were adapted from Derryberry and Reed’s (2002) Attentional Control Scale (ACS) and Barry et al.’s (2013) Emotional Attentional Control Scale (eACS). These questions covered three dimensions of control: attention focus, shift, and emotional attention control, with Questions 1–3 on attention focus control, Questions 4–6 on attention shift control, and Questions 7–9 on emotional attention control. This part of the questions was labeled Attention Control Scale (ACS).

It is necessary to note that some of the IMS and ACS questions were negatively stated (e.g., IMS Question 5 “English writing gives me a lot of pressure. I do not enjoy English writing”). The ratings on these questions were reversed in the data analysis for the results to be meaningful.

#### Data collection and analysis

The 216 participating students were asked to complete the four-part questionnaire online using mobile devices at the end of the English Academic Writing course. Then we also obtained these students’ course grades/scores from the instructors. It is necessary to note that the data of 30 students were excluded for one of the following two reasons. Around 17 were excluded because of the same problems mentioned in the preliminary study, i.e., these students randomly responded to the questionnaire by selecting either the same answer or the same pattern of answers throughout the questionnaire. The other 13 were excluded because they did not complete their writing assignments and hence had not course grade or score. In short, the number of participants whose data were included in the analysis was 186.

For data analysis, as was done in answering Question 1 in the preliminary study, we first ran a Cronbach’s Alpha test of all three parts of the questionnaire to ascertain their reliability. The results show acceptable Alpha reliability levels for all the three parts (0.858, 0,806, and 0.806, respectively). Then, to answer research Question 3 (“Whether and how frequently do EFL students experience flow in writing across a semester-long writing course?”), we employed the LCFA procedure to uncover the flow frequency profiles of the students. To answer research Question 4 (“What is the relationship between EFL students’ flow experience and their perceived level of intrinsic motivation and attention control?”), a Spearman’s rank correlation test was conducted to explore the relationship. To answer research Question 5 (“What is the effect of students’ flow frequency on their writing performance?”), a simple linear regression test was conducted to determine whether flow frequency affected or predicted students’ writing course performance (course grade/score).

### Results

The goodness-of-fit statistics of the LCFA concerning the various possible groupings (models) of the students based on their response scores on the *WFFS* (reported in Table 5) show seven models for grouping the participating. Of the seven models, model 4 (with four groups) had the lowest BIC value (4142.64) and hence was the best-fit model.

**Table 5:** Goodness-of-Fit statistics for LCFA models on *WFFS* (*N* = 186).

Model	LL	BIC(LL)	Npar	df	p value
1 Group	−2216.512	4621.150	36	150	6.1e−422
2 Groups	−2031.787	4303.959	46	140	1.0e−352
3 Groups	−1951.562	4195.765	56	130	4.8e−326
4 Groups	−1898.871	4142.640	66	120	1.4e−310
5 Groups	−1872.850	4142.857	76	110	5.10E−306
6 Groups	−1848.020	4145.455	86	100	5.00E−302
7 Groups	−1828.705	4159.081	96	90	2.00E−300

**Figure 2:**
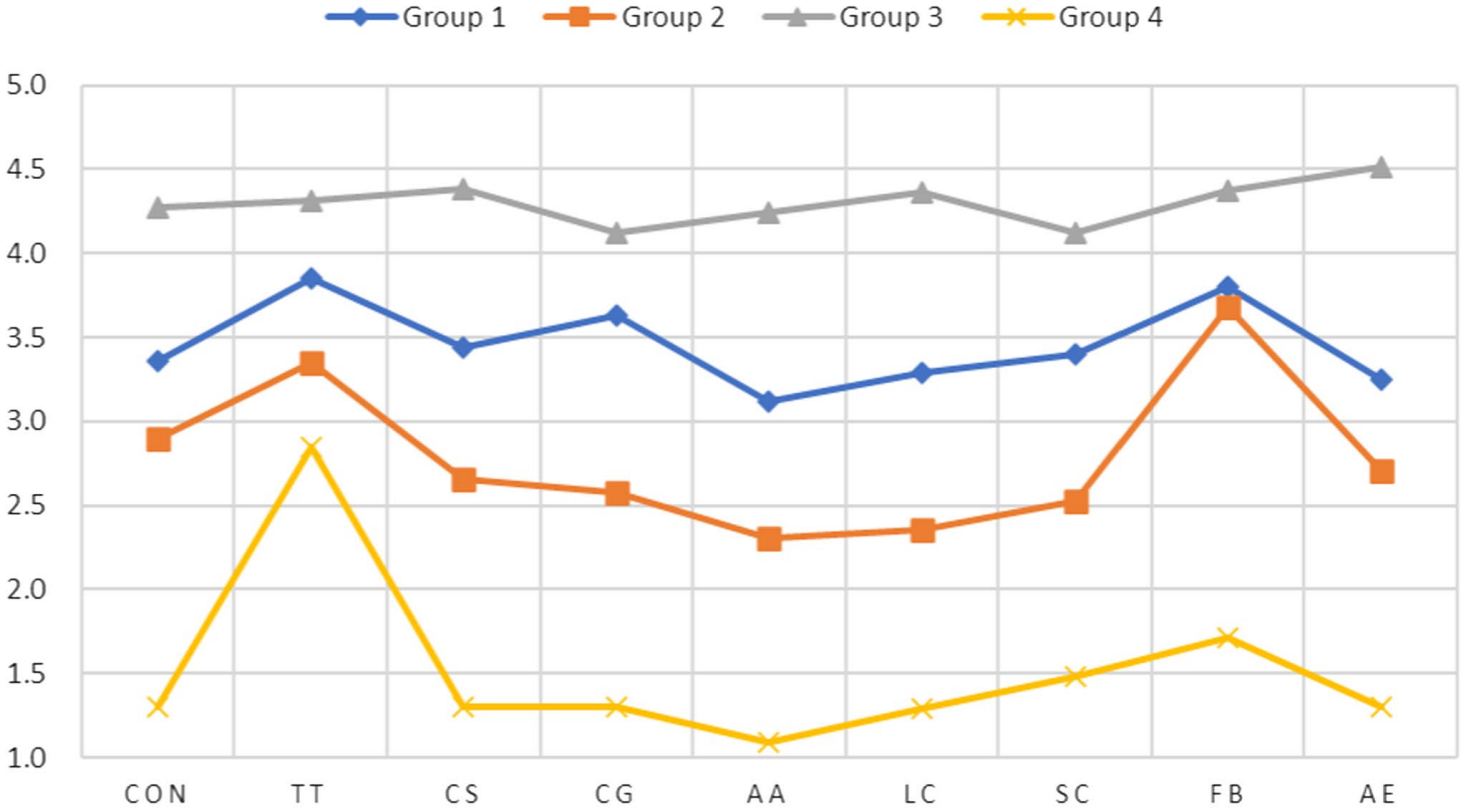
Profile plot of four-group LCFA Model of *FFE*. CON, concentration on the task at hand; TT, transformation of time; CS, challenge-skill balance; CG, clear goals; AA, action-awareness merging; LC, loss of self-consciousness; SC, sense of control; FB, unambiguous feedback; and AE, autotelic experience. The *y*-axis listed item-average scores and the *x*-axis presented flow dimensions.

After it was determined that the four-group model was the best fit, we were then able to find out the four groups’ response pattern profiles. The LCFA yielded the following types of information regarding the groups’ profiles: (1) the percentage of each of the four groups in the total number of participants and (2) each group’s mean score in each of the nine dimensions of flow frequency measured by the *WFFS.* The four groups’ profile information is reported in Table 6. A profile plot based on this information is presented in Figure 2.

**Table 6:** Profile of the Four-Group LCFA Model of *WFFS.*

Category	Subcategory/Dimension	Group 1	Group 2	Group 3	Group 4
Percentage		0.518	0.308	0.146	0.028
*PFFS* Perceived flow frequency scale	Concentration	3.355	2.896	4.276	1.301
	Transformation of Time	3.851	3.353	4.312	2.849
	Challenge-Skill Balance	3.440	2.652	4.379	1.300
	Clear Goals	3.629	2.580	4.120	1.302
	Action Awareness Merging	3.122	2.309	4.245	1.095
	Loss of Self-consciousness	3.290	2.355	4.361	1.291
	Sense of Control	3.401	2.526	4.122	1.486
	Unambiguous Feedback	3.802	3.682	4.377	1.711
	Autotelic Experience	3.245	2.704	4.519	1.297
	**Total mean**	**3.459**	**2.784**	**4.301**	**1.515**

The bold figures are the total numbers. Bold is used to help differentiate the total numbers from the other numbers.

As shown in Table 6, Groups 3 boasted the highest overall flow frequency mean (4.301) with a mean above 4 in each of the nice flow dimensions, followed by Group 1 (3.459) with a mean above 3 in all nine dimensions while Groups 2 and 4 each showed an overall flow frequency mean below 3 (2.784 and 1.515, respectively) even though Group 2 had a mean above 3 in two dimensions. Furthermore, both Groups 1 and 3 showed a mean score above 3.4 in all the three of the dimensions (challenge-skill balance, Clear goals, and Unambiguous feedback) where a 3.4 scale was the benchmark for indicating the experience of flow (Kawabata and Mallett, 2012). Given that a scale of 1 in *FFS* meant Never and a 5 meant Always (with 2 = rarely, 3 = Occasionally, and 4 = frequently) and that a mean score had to be above 3 to indicate the occurrence of flow (Kawabata and Mallett, 2012), the results seem to suggest that Group 3 experienced flow very frequently and Group 1 experienced it sometimes or occasionally while Groups 2 and 4 rarely or almost never experienced it, making the latter two groups non-flow groups. The results also indicate that 66.4% of the students (Group1’s 51.8% + Group 3’s 14.6%) experienced flow with a frequency ranging from low (occasionally) to high (frequently) while 33.6% (Group 2’s 30.8% + Group 4’s 2.8%) rarely or never experienced flow.

The results of the Spearman’s rank correlation test (reported in Table 7) reveal significant correlations among the students’ level of intrinsic motivation, attention control, and flow frequency. This means that when students’ level of intrinsic motivation and attention control ability increased, so would their flow frequency or vice versus. In other words, there appears to be a virtuous circle among the three psychological states or variables, i.e., they promote one another, a point that we will explore in the Discussion section below.

**Table 7:** Spearman’s rank correlations between.

	WFFS	IMS	ACS
*WFFS*	1.000	0.614^**^	0.414^**^
*IMS*	0.614^**^	1.000	0.369^**^
*ACS*	0.414^**^	0.360^**^	1.000

**indicates a statistical significant correlation at *p* < 0.01.

The results of the simple linear regression test indicate that the students’ flow frequency explained 6.6% of the variance in their writing scores [*R^2^* = 0.066, *F*(1, 185) = 12.926, *p* < 0.000]. It was found that the students’ flow frequency significantly predicted their writing course final scores (*β* = 1.746, *p* < 0.000), i.e., when a student’s flow frequency increased one unit, his/her writing score would increase 1.746 points. This finding is consistent with those of previous research showing that a higher level of flow intensity and a higher flow frequency may induce frequent practice, more engagement, and better performance in what students are learning (O’Neill, 1999; Shernoff et al., 2003).

## Discussion

In discussing the findings, a recapitulation of the main results of our two studies is first in order. As shown above, both the preliminary study and the main study confirmed that a majority of the EFL writers did experience flow in L2 writing to various degrees of intensity and frequency. Furthermore, the results of the regression analysis in the preliminary study show that students with a higher perceived level of writing skills were more likely to experience flow in writing than those with a low perceive level of skills and there also appeared to be a higher (though not statistically significantly higher) tendency for students with a lower perceived level of challenges in the writing task to experience flow than those with a higher perceived level of challenges. Similarly, the Spearman’s rank correlation test in the main study reveals that students’ intrinsic motivation levels and attention control levels were significantly correlated with their flow frequencies in writing. Finally, the results of the simple linear regression test in the main study indicate that students’ flow frequency can significantly influence their writing performance, i.e., when students’ flow frequencies increase, their writing course scores would also rise.

To better understand what all the results from out two studies mean, we need to first look at what experiencing flow in writing really means. Based on the nine dimensions of writing flow, students who experience flow, especially those showing a high level of flow intensity and frequency, will have a strong concentration on the writing task, clear writing goals (purposes), a loss of self-consciousness, a feeling that their writing skills match the challenges of the writing task (i.e., a challenge-skill balance), and an unusual sense of time passing, all of which contribute to a feeling of smooth writing. In short, writing in a flow state is a very positive experience that makes the writing process enjoyable and smooth.

Given the significant positive correlations between flow experience and other psychological factors, such as intrinsic motivation and attention control, and given the virtuous circle these variables appear to be in, we could reasonably expect flow experience to lead L2 writers to a higher inner interest and intrinsic motivation in writing, a better sense of achievement, more engagement in writing tasks, and a greater satisfaction in their writing products. In fact, some empirical research (e.g., Abbott, 2000) has confirmed such virtuous effects of flow on writing. In addition, when students have increased intrinsic motivation and flow experience, they would be more active and proactive with their writing assignments, avoiding procrastination (Lee, 2005), as well as be better able to resist distractions and in turn perform better in writing (Camacho et al., 2021). Similarly, experiencing flow frequently may enable students to keep their emotions positive and turn negative emotions into positive energies (Chen et al., 1999; Moneta and Csikszentmihalyi, 1999).

Most importantly, as shown in the main study, flow frequency also has a significant positive influence on writing performance. Hence it is pivotal for L2 writing teachers to help their students to experience flow as intensely and frequently as possible during writing. Then comes the most crucial and challenging question of how. To answer this question, we should understand what psychological factors and teaching practices may help lead L2 writers to flow experience. As shown in the preliminary study, a higher perceived level of writing skills along with a lower perceived level of challenges of the writing task tend to help students experience flow. Furthermore, the results of the main study indicate that students with a higher level of intrinsic motivation and attention control also reportedly experience a higher flow frequency. In terms of teaching practices, previous research has shown that familiar writing tasks along with the use of familiar technology tend to enable L2 writers to experience flow in writing (Payant and Zuniga, 2022) and that multimodal and multiliteracy assignments are more likely to generate flow experience in ESL writers than traditional mono-modal and mono-literacy assignments (Ringel, 2014).

These research findings indicate clearly the importance for L2 writing students to develop a strong intrinsic motivation and good attention control ability. To develop a strong intrinsic motivation for writing, L2 writers will need to appreciate the importance of excelling in the writing tasks they are undertaking, feel an enjoyment in doing their writing, and understand how their writing may help them in their future lives and work (Graham, 2018). To help students in this regard, empirical research (e.g., Yeung et al., 2020, p. 443) has shown that teachers should “facilitate the integration of the external values of writing into the goals that have significance” for students. Furthermore, teachers should design writing assignments that are meaningful and engaging to students (Ringel, 2014). Regarding helping students developing a better attention control ability, teachers may try having their students practice some attention enhancement activities, such as practice various forms of meditations that call for “a deliberate focus of attention on certain aspects of present moment-to-moment experience, monitoring of distractions, and reorienting toward the object of attention in the meditation” (Trautwein et al., 2020, p. 5). Attention training techniques, such as “selective attention,” “attention switching,” and “divided attention” (i.e., focusing on more than one thing), have been proven effective in research (Fergus and Limbers, 2019, p. 804). Last and perhaps most importantly, teachers should try to design writing assignments on topics which students are interested in and familiar with so they would feel more engaged and also more confident in what they are writing, which would in turn lead them to experiencing flow (Payant and Zuniga, 2022). Whenever possible, assignments should also involve multi-modalities and multi-literacies (Ringel, 2014).

## Conclusion

### Main findings and implications

*Via* two questionnaire-based studies with large sample sizes, this research has investigated L2 writers’ flow experience in terms of intensity and frequency and the relationship of L2 writers flow experience with their other psychological states and writing performance. The results of the study have demonstrated that (1) many L2 writers experience flow to various degrees of intensity and frequency during writing, (2) flow experience is significantly correlated with some other important psychological states (such as perceived writing challenges and skills, intrinsic motivation, and attention control), hence forming a virtuous circle with the latter, and (3) flow experience has a significant positive influence on writing performance. Based on the characteristics of flow experience, when students are in flow, they would have, among other things, a heightened concentration on the writing task with clear writing goals and better abilities to resist distractions, a strong intrinsic interest and motivation in performing the writing task, high confidence, a smooth and enjoyable writing process, and a successful writing performance.

In short, this study probed into the influence of flow experience on L2 writing performance and its relationship with L2 writers’ psychological states during writing. The findings also highlighted the importance of flow experience in enhancing L2 writers’ writing experience and reducing their negative emotions toward writing. These findings have both important research and pedagogical implications, to which we now turn.

Given the importance of flow experience in L2 writing, it is thus imperative for teachers to help L2 writers experience flow in writing by creating a supportive learning environment, promoting the development of the psychological states that are inductive to flow experience, and design engaging writing assignments that not only motivate students but also present the level of challenges that match students’ level of writing skills. More specifically, L2 teachers may do the following to help L2 writers experience flow and enhance their writing performance. First, teachers can regularly ascertain their students’ perceived writing skills, challenges, and interests *via* brief surveys, discussion groups, and informal conversations with students. Based on the feedback from students, teachers can design writing assignments that are within their students’ range of skills and are of interest to the students. Second, to induce flow experience in writing, teachers can adopt attention training techniques to help students learn to better focus their attention on their writing tasks, such as those techniques mentioned above including “deliberate focus,” “selective attention,” “attention switching,” and “divided attention” (Fergus and Limbers, 2019; Trautwein et al., 2020). Third, to increase students’ interest and motivation in writing, teachers can develop writing assignments that are directly related to or needed in their students’ future work so students can better appreciate the value of the writing tasks they perform. Fourth, because flow experience in writing often varies across students in intensity and frequency, teachers will need to make adjustments in assignments and instruction tailored to individual students’ needs so students can better capitalize on their own strengths and overcome their weaknesses.

### Limitations and future research

The data obtained *via* the questionnaires in this study were self-reported; as such, they were not highly reliable for the assessment of psychological states. This is because the participating students might have exaggerated or understated their true feelings either consciously or unconsciously because of their unfamiliarity of the scale or some other factors, such as being judged. Furthermore, due to varying response styles and individual differences, some students would favor extreme answers while others are likely to choose neutral ones. Another major limitation of this study was that survey data were the only type of data. No interview and observation data were collected, making the results of the study untriangulated and hence not very reliable. One more limitation of this study was that it included only Chinese college EFL students. Hence, the findings may not apply to L2 learners of other age, L1, and education backgrounds.

For future research, scholars should take the above issues into consideration to enhance the validity and reliability of their questionnaire data. More importantly, they should try to include other forms of data, such as interviews and observations during the writing process (either human observations or machine-based ones, such as eye-tracking, or both types if feasible). Furthermore, future research on flow in L2 writing may need to include L2 writers of different L1s, age groups, and education levels. Researchers may also investigate whether there are differences in flow experience between the two genders. In addition, more research needs to be done on developing flow metrics used specifically for assessing flow experience in writing, especially L2 writing, rather than using and/or adapting flow metrics from other fields, such as sports, as has often been the case thus far.

## Data availability statement

The raw data supporting the conclusions of this article will be made available by the authors, without undue reservation.

## Ethics statement

Ethical review and approval was not required for the study on human participants in accordance with the local legislation and institutional requirements. The patients/participants provided their written informed consent to participate in this study.

## Author contributions

All authors listed have made a substantial, direct, and intellectual contribution to the work and approved it for publication.

## Conflict of interest

The authors declare that the research was conducted in the absence of any commercial or financial relationships that could be construed as a potential conflict of interest.

## Publisher’s note

All claims expressed in this article are solely those of the authors and do not necessarily represent those of their affiliated organizations, or those of the publisher, the editors and the reviewers. Any product that may be evaluated in this article, or claim that may be made by its manufacturer, is not guaranteed or endorsed by the publisher.
