# Supplementary material for: Flow experience in foreign language writing: Its effect on students’ writing process and writing performance
Source: Front Psychol. 2022 Aug 4;13:952044. doi: 10.3389/fpsyg.2022.952044 (PMC9387668; doi:10.3389/fpsyg.2022.952044)
Supplement: Supplementary file 1 [file Data_Sheet_1.pdf]

Note: English translation is provided in parentheses after each Chinese item

Appendix 1

大学生英语写作心理状态调查 (Questionnaire Regarding College Students' Psychological States Druing Writing)

基本信息：(Basic demographic information)

1) 学校：(College/School in the University)
2) 年级：(Year of College Study)
3) 班级： (Class)
4) 学号：(Student ID number)
5) 性别：(Gender)

Part 1

1. 请根据刚才的写作体验回答以下问题： (Answer the following questions based on your experience in the writing you just completed)
2. 请对刚才的写作任务进行评分（1-10 分）(Rate your own writing performance using a scale of 1-10) ：
1. 请对刚才的写作难度进行评分：(Rate the difficulty level of the writing task)
2. 请对自身写作能力进行评价：(Rate your own writing ability)

Part 2: Writing Flow State Scale (WFSS)

在刚才的写作过程中 (1-很不同意；5-很同意)：(Based on your experience, rate your level of agreement with the following statements regarding your actions/feelings during the writing you just completed, using the following sale: 1=strongly disagree; 5=strongly agree)

1. 我的注意力完全集中于写作中，没有分心。(My attention was focused completely on the writing with no distraction)
2. 我感觉写作过程中时间好像比平时过得更快。(I felt that during the writing time went faster than usual)
3. 我觉得刚才的写作任务对我来说难度刚刚好。(I felt the level of difficulty of the writing task was just right)
4. 我知道自己想表达什么观点，想写出什么样的文章。(I knew what I wanted to express and what kind of essay I wanted to produce)
5. 我感觉刚才的写作过程很顺畅。(I felt the writing just completed went very smoothly)
6. 我完全沉浸在写作中，不在意别人有没有注意到我的写作过程。(I was totally engaged in the writing task, paying no attention to whether others noticed my writing process)
7. 我能把控整个写作过程，能够决定表达什么观点、怎么表达观点、怎么架构文章结构。(I had control over the entire writing process, was able to decide what opinions to express, how to express them, and how to organize my essay.)
8. 从我的写作过程我大概知道自己写得怎么样。(Based on my experience in the writing process, I knew roughly how well I wrote)
9. 我愿意再继续进行类似的写作活动。 (I want to continue doing this type of writing)

Appendix 2

大学生英语写作心理状态调查(Questionnaire Regarding College Students' Psychological States druing Writing)

基本信息：(Basic demographic information)
1) 学校：(College/School in the University)
2) 年级：(Year of College Study)
3) 班级：(Class)
4) 学号：(Student ID number)
5) 性别：(Gender)

请根据本学期的写作体验回答以下问题：(Answer the following questions based on your writing experience during the semester)

Part 1: Writing Flow Frequency Scale (WFFS)

以下问卷为测量日常写作心理状态，请根据您本学期的写作任务和写作过程中的真实体验选择对应的选项：(The following questions aim to assess your usual psychological states in writing during the semester. Rate your level of agreement/disagreement with the following statements regarding the frequency of the actions/feelings mentioned based on your true experience over the semester)

请根据发生以下情况的频率进行评分（1-从不；5-总是）(1=never; 5=always)：

1. 在进行英语写作的时候，我的注意力完全集中于写作中，没有分心。(During writing, my attention is focused completely on the writing with no distraction)
2. 在英语写作过程中，我感觉时间好像比平时过得更快。(During the writing process, I feel time goes faster than usual)
3. 我觉得本学期的英语写作任务对我来说难度刚刚好。(I feel the level of difficulty of the writing tasks this past semester was just right)
4. 在英语写作过程中，我通常知道自己想表达什么观点，想写出什么样的文章。(During writing, I usually know what I want to express and what kind of essay I want to produce)
5. 在本学期的英语写作过程中，我的写作过程很顺畅。(During the semester, my writing all went very smoothly)
6. 英语写作时，我完全沉浸在写作中，不在意别人有没有注意到我的写作过程。 When writing, I am totally engaged in the writing task, paying no attention to whether others notice my writing process)
7. 我能把控整个英语写作过程，能够决定表达什么观点、怎么表达观点、怎么架构文章结构。(I have control over all my writing process, am able to decide what opinions to express, how to express them, and how to organize my essay.)
8. 从我的英语写作过程我大概知道自己写得怎么样。Based on my experience in the writing process, I know roughly how well I write)
9. 我愿意再次进行类似本学期的英语写作任务。(I want to continue doing the types of writing we did this semester)

Part 2: (Intrinsic Motivation Scale) IMS

1. 我每周都会通过阅读英语文章或者看英语影视剧等不同方式来积累写作素材。(Every week, I read English articles, watch English movies/TV drama, as well as doing other English activities to gather materials for writing)
2. 我希望我可以把英语写作中学到的语言知识运用到生活中。(I hope I can apply the language skills I learn from writing to everyday life)
3. 比起老师的修改意见，我更在意自己英语作文成绩高低。(Compared with teachers' assessment, I mind more my own feelings about my writing.
4. 即使老师没有布置写作任务，我平时也会主动写英文文章（或记英文日记）。(Even if

teachers do not give us writing assignments, I usually write essays or diaries myself)

5. 我总是认真完成每一次英语写作作业。(I always diligently complete every writing assignment)

6. 我更愿意接受有挑战性的英语写作任务，如用复杂的句型或高级词汇写作等。(I prefer more challenging writing tasks, for example using more complex sentence structures and vocabulary in writing)

7. 英语写作给我很大的压力，我不享受写作的过程。(English writing places a lot of pressure on me. I do not enjoy the writing process)

8. 我希望我的英语作文可以说服别人或者给人启发。(I hope my English writing can persuade others or enlighten others).

9. 我写英语作文是因为完成作文之后心里会有成就感。(I do English writing because I have a feeling of an accomplishment every time I complete a writing task)

10. 我会尽可能逃避英语写作任务。(I always try not to do writing assignments)

11. 我写英语作文是因为老师的要求，英语写作并不能让我学到新知识。(I am doing English writing simply because my teacher requires me to. English writing cannot help me learn new knowledge.

Part 3: Attention Control Scale (ACS)

当我在写作时（1-很不同意；5-很同意）：(When I am writing, 1= strongly disagree; 5=strongly agree)

1. 即使周围有噪声/杂音，我也能很好地集中注意力写作。(Even if there are a lot of noises, I can still focus my attention on writing)

2. 当我认真写作时，还是容易被周围发生的事情干扰。(When I am writing, I easily become disturbed or distracted by what is going on around me)

3. 当我尝试集中注意力写作的时候，我的脑子里会想到其他无关的事。(When I try to focus my attention on writing, I often think about other things unrelated to the writing task)

4. 当我被周围的事情干扰，我可以快速地将我的注意力转移回写作上。(When there is an outside distraction, I can also quickly shift my attention back to my writing task)

5. 当我在写作过程中出现分心的想法时，我可以快速将注意力抽离这些想法。(When I become distracted during writing, I can quickly shift my attention away from the distraction)

6. 我要花很长时间才能真正进入写作状态。(It takes a long time for me to truly get into doing my writing)

7. 写作时，如果我情绪不稳定，我的注意力很容易转移到别的事情上。(When I am writing, if my emotions fluctuate, my attention will easily go to other things)

8. 如果写作时情绪烦躁的话，我很难集中注意力。(If I feel frustrated during writing, I cannot be concentrated)

9. 我不会把自己的情绪带入写作过程，我的情绪不会影响我的写作结果。(I will not let emotions get into my writing. My feelings will not affect my writing performance)
